# Response of spinal myoclonus to a combination therapy of autogenic training and biofeedback

**DOI:** 10.1186/1751-0759-1-18

**Published:** 2007-10-12

**Authors:** Koreaki Sugimoto, Theoharis C Theoharides, Duraisamy Kempuraj, Pio Conti

**Affiliations:** 1 Division of Psychosomatic Medicine, Preventive Welfare Clinic, Tohoku Fukushi University, Sendai, Japan; 2 Department of Internal Medicine, Tufts-New England Medical Center, Boston, USA; 3 Department of Pharmacology and Experimental Therapeutics, Tufts University School of Medicine, Boston, USA; 4 Department of Immunology, University of Chieti, School of Medicine, Chieti, Italy

## Abstract

**Introduction:**

Clinical evidence indicates that certain types of movement disorders are due to psychosomatic factors. Patients with myoclonic movements are usually treated by a variety of therapeutic agents. Autogenic training (AT), a recognized form of psychosomatic therapies, is suitable for certain types of neurological diseases. We describe a patient with myoclonus who failed to respond to conventional medical therapy. His symptoms were exaggerated by psychogenic factors, especially anger.

**Case presentation:**

A 42-year-old man was admitted to our hospital, Preventive Welfare Clinic, for severe paroxysmal axial myoclonus of the left shoulder and abdominal muscles. The initial diagnosis was "combination of spinal segmental myoclonus and propriospinal myoclonus". The myoclonic movements did not occur during sleep but were aggravated by bathing, alcohol drinking, and anger. Psychological examination indicated hostile attribution. Although considered not to be a case of psychogenic myoclonus, a "*psychogenic factor*" was definitely involved in the induction of the organic myoclonus. The final diagnosis was "combination of spinal segmental myoclonus and propriospinal myoclonus accompanied by features of psychosomatic disorders". The patient underwent psychosomatic therapy including AT and surface electromyography (EMG)-biofeedback therapy and treatment with clonazepam and carbamazepine.

**Results:**

AT and EMG-biofeedback resulted in shortening the duration and reducing the amplitude and frequency of the myoclonic discharges.

**Conclusion:**

Psychosomatic therapy with AT and surface EMG-biofeedback produced excellent improvement of myoclonic movements and allowed the reduction of the dosage of conventional medications.

## Introduction

There is clinical evidence that indicates that certain types of movement disorders are due to not only organic lesions but also related to psychosomatic factors [1-5]. Psychosomatic disorders (PSD) is a term used to describe a syndrome in which symptoms are aggravated by psychological factors. Patients with myoclonic movements are usually treated by a variety of therapeutic agents such as anticonvulsants and benzodiazepines because of their sedative effects [6-12]. On the other hand, autogenic training (AT) is considered an effective therapy for patients with hypertension, insomnia, headache, Raynaud's disease, skin disorders, anxiety [13] and pain [14]. In neurological diseases, one case report showed that myoclonus secondary to anoxic encephalopathy was reduced by autogenic relaxation and electromyography (EMG) biofeedback [15]. This case is the second report of successful improvement of myoclonus by AT.

A patient with myoclonus was referred to our hospital, Preventive Welfare Clinic, from another hospital after failure to respond adequately to medical therapy. We did a combination treatment of AT and biofeedback. Surface EMG during AT was recorded to monitor myoclonic discharge. AT-surface EMG therapy can provide a visual negative biofeedback effect (EMG-Biofeedback). In this report, we describe a patient with spinal myoclonus in whom AT-EMG-Biofeedback combination therapy resulted in excellent improvement of symptoms.

### Case Presentation

A 42-year-old man was admitted to our hospital for severe paroxysmal axial myoclonus of the left shoulder and abdominal muscles. The patient had been quite well and was a member of a self-defense force team until he developed spontaneous involuntary movements about 20 times per minute. The myoclonic movement gradually involved muscles of the left trunk, left lower extremity and right extremities within one month. The myoclonic movement did not occur during sleep but worsened upon taking a bath, drinking alcohol, and whenever the patient was angry. The patient was first admitted to a University hospital and diagnosed as "combination of spinal segmental myoclonus and propriospinal myoclonus" [16]. The patient was treated with clonazepam (3.5 mg/day) and carbamazepine (400 mg/day). However, this treatment had no major effect on the symptoms. The condition worsened after discharge. Tapping anywhere on the body, including the face and spine, with a neurological hammer induced myoclonic contractions. The patient visited another medical clinic because of dissatisfaction with the medical treatment. A few years later, the patient was referred to our hospital, Preventive Welfare Clinic, for consultation.

On admission, physical and neurological examinations showed no abnormal findings apart from the myoclonic involuntary movements. Routine laboratory tests and MRI study of the cervical and thoracic spinal cords showed no abnormal findings. The myoclonus consisted of continuous rhythmic muscle contractions of the thoracic and abdominal muscles bilaterally, with left-side predominance. Tapping on certain points on the body, for example the left pectoralis major muscle or spine, with a neurological hammer induced myoclonic discharge. However, sound or light stimulation and elicitation of tendon reflex did not induce the myoclonic discharge. Medical history was negative for major accidents such as trauma and fever, and there was no change of working circumstances after the patient developed the myoclonic movements. Based on the description of anger-elicited worsening of myoclonic movements, the patient underwent a battery of psychological tests. The hysteria scale on the Minnesota Multiphasic Personality Inventory (MMPI) was very high (T score = 78). In the Cook-Medley Hostility Scale, the hostility and cynicism scales were high (above the average scale + SD). The score on the Cornel Medical Index (CMI) was within normal range, but the hostility scale was high.

Although clonazepam, carbamazepine, and other sedative medications were used at relatively high doses, the treatment failed to control the myoclonic movements. While it was concluded that the myoclonus was due to organic disease, anger/rage was associated with worsening of symptoms and the patient seemed to have a hostile character. Psychogenic myoclonus is known as a cause of movement disorders. Although this patient was not considered a case of psychogenic myoclonus, a "*psychogenic factor*" was definitely involved in the induction of the myoclonus. Our final diagnosis was a "combination of spinal segmental myoclonus and propriospinal myoclonus accompanied by features of psychosomatic disorders". Accordingly, we tried psychosomatic therapy; a combination of AT and EMG-biofeedback therapy, to reduce myoclonic movement and drug dosage.

## Methods

### Autogenic Training (AT)

AT was used for therapy as described previously [17]. First, we described the significance of the AT effect, adaptation to AT, and how to perform AT, using a cassette tape. The patient started AT for 5 min, three times every day without changing the medications.

### EMG-Biofeedback

**Figure 1:**
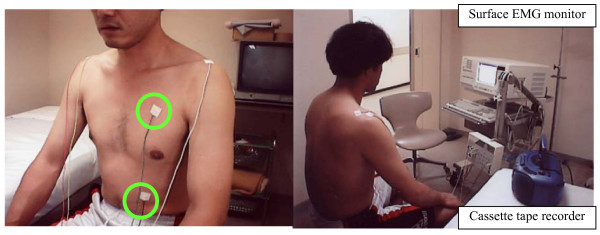
**Photograph of surface EMG monitoring during autogenic training (AT)**. The patient was prepared by placing the electrodes on the left major pectoral and abdominal rectal muscles (green circles). The references and ground electrodes were placed on the right and left shoulder, respectively. Patient did AT training using a cassette tape recorder for 5 min, 3 times a day. During AT, the patient could watch the myoclonic jerks on the surface EMG recorder, which served as a biomechanical feedback.

Surface EMGs of the left major pectoral muscle and abdominal rectal muscle were recorded during AT training in the hospital (Figure 1). The severest myoclonic movements were noticed in the left major pectoral muscle and abdominal rectal muscle. The EMG data were recorded before and during the 5-min AT performance, and the records were shown to the patient during feedback cognition. The recording of EMG-Biofeedback was done just before AT training and 2, 6, 9, 13, and 16 days after AT training. In total, AT and EMG-Biofeedback therapy were performed for about 15 min, twice a week.

## Results

**Figure 2:**
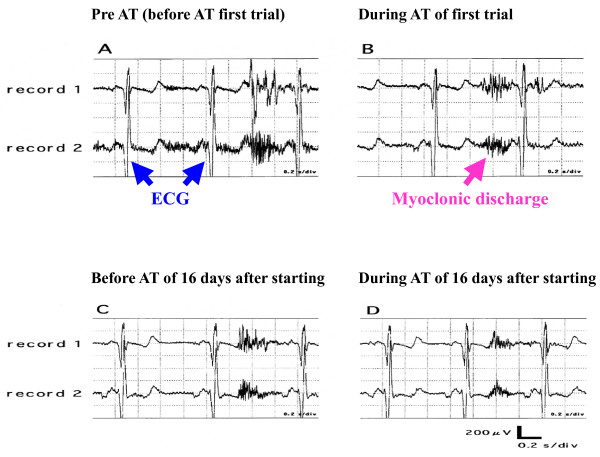
**Representative records of surface EMG**. Representative records of surface EMG from the left major pectoral (record 1) and left abdominal rectal muscles (record 2). Records of first trial of AT appear as Pre AT (A: before AT first trial) and During AT (B). Records after 16 days of AT training are marked as Before AT (C) and During AT (D). Myoclonic discharges are shown in red. Both the duration and amplitude of myoclonic discharges were decreased by AT after 16 days of AT training.

**Figure 3:**
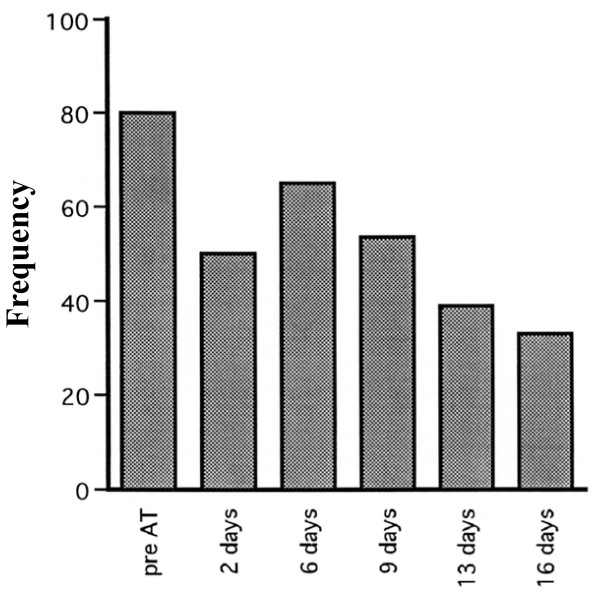
**Frequency of myoclonus in 5 min**. Frequency of myoclonus discharges within 5 min decreased progressively and significantly from 80 to about 34 at day 16 after the commencement of AT training.

Figure 2 shows the raw data of the surface EMG. The duration and amplitude of myoclonic discharge during AT were different from those of baseline (before AT). The duration of myoclonic discharge during AT was shorter than that before AT in the first trial and also during the trial at day 16 after commencement of AT training. Furthermore, the amplitude recorded during AT was smaller than that at before AT in the first trial and also during the trial at day 16, after the commencement of AT training. These results indicate that myoclonus discharge decreased during AT. Comparison of AT on the first trial and at day 16 after AT training showed that both duration and amplitude of the discharge were shorter and smaller in the latter, indicating that AT training for 16 days decreased the myoclonus. Table 1 shows the duration (sec) and amplitude (μV) of the myoclonic discharge before and at day 16 after AT as recorded from the left major pectoral muscle and abdominal rectal muscle. The duration and amplitude of myoclonic discharge in both muscles were significantly lower at day 16 after the commencement of AT training. The frequency of myoclonic discharge recorded within 5 min also decreased progressively and significantly from 80 to about 34 at day 16 after the commencement of AT training (Figure 3).

## Discussion

Psychogenic myoclonus is a recognized etiology of movement disorders. The characteristic features of psychogenic myoclonus are: 1) clinical features incongruous with organic myoclonus, 2) evidence of underlying psychopathology, 3) improvement with distraction, and 4) the presence of incongruous sensory loss or false weakness [18]. Since our case was not consistent with criteria 2 and 4 above, this patient was not diagnosed as psychogenic myoclonus. In fact, this patient did not show spontaneous remission and placebo response. However, the myoclonus was worsened by bathing, alcohol consumption, and anger, and it disappeared during sleep. When psychogenic factors are suspected of influencing the symptoms, a psychosomatic approach should be considered as an optional therapy. In our case, we were able to reduce the dosages of medications from 3.5 mg/day of clonazepam and 400 mg/day of carbamazepine on admission to 2 mg/day of clonazepam only on discharge. After repetitive AT training and EMG-biofeedback therapy, the patient emphasized that he became more calm and that he was able to control his emotions. This awareness must have been significant to him.

Only one report has described the use of autogenic training with EMG biofeedback therapy and that such a protocol reduced the myoclonus, which was induced by anoxic encephalopathy [15]. Though myoclonus is a neurological disease, a psychosomatic approach should be considered as an optional therapy, especially for patients with psychogenic factors known to enhance the symptoms.

## Conclusion

Our patient with spinal myoclonus responded well to the combination therapy of AT and surface EMG-Biofeedback. Such therapy reduced the duration, amplitude, and frequency of myoclonic jerks and, consequently, we were able to reduce the dosage of clonazepam and carbamazepine.

## Abbreviations

Autogenic training (AT), Electromyography (EMG)

## Competing interests

The author(s) declare that they have no competing interests.

## Authors' contributions

KS treated the patient as the chief medical doctor. TCT and KS designed the study, analyzed the data, and wrote the manuscript. DK and PC gave advice on important techniques and discussion. All authors read and approved the final manuscript.

**Table 1:** Duration and amplitude of myoclonic discharge during AT performance

	Left major pectoral muscle		Left abdominal rectal muscle	
	Pre AT	16 days	Pre AT	16 days
		
Duration (sec)	0.61 ± 0.07	0.41 ± 0.06*	0.61 ± 0.10	0.27 ± 0.08*
Amplitude (μV)	1163 ± 410	283 ± 145*	862 ± 285	159 ± 56*

Pre-AT: myoclonic discharge before AT training on the first trial

16 days: myoclonic discharge after AT training

Data are mean ± SD of all myoclonic discharges recorded within 5 min during AT.

*p < 0.001, compared with Pre AT (unpaired T test).
